# Facilitators and Barriers to Implementing VR in Dementia Care Training for Formal Caregivers: A Scoping Review

**DOI:** 10.1093/geroni/igaf122.2661

**Published:** 2025-12-31

**Authors:** Lillian Hung, Carol Hok, Ka Ma, Chih Yun Huang, Joey Oi, Yee Wong, Karen Lok, Yi Wong, Keng Hao Chew, Lily Haopu Ren, Yong Zhao

**Affiliations:** University of British Columbia, Vancouver, British Columbia, Canada; Singapore University of Social Science, Singapore, Singapore; National Taiwan University Hospital, Taipei, Taipei, Taiwan (Republic of China); University of British Columbia, Vancouver, British Columbia, Canada; The University of British Columbia, Vancouver, British Columbia, Canada; Nanyang Technological University, Singapore, Singapore; The University of British Columbia, Vancouver, British Columbia, Canada; The University of British Columbia, Vancouver, British Columbia, Canada

## Abstract

As the aging population grows, the need for improved dementia care training for formal caregivers is urgent. Virtual reality (VR) offers a promising approach to enhance training outcomes. This scoping review examines the facilitators, barriers, and impacts of implementing fully immersive VR in dementia care training for formal caregivers in long-term care settings. Guided by the Consolidated Framework for Implementation Research, this review followed the Joanna Briggs Institute methodology and PRISMA-ScR guidelines. A systematic search of CINAHL, MEDLINE, Embase, Scopus, Web of Science, and ProQuest identified 469 publications, with nine meeting inclusion criteria. These studies, published between 2015 and 2024, involved 362 formal caregivers aged 44.7 to 65 years. VR interventions fostered empathy through first-person perspectives and helped participants recognize behavioral triggers and apply caregiving strategies using second- and third-person perspectives. Barriers and facilitators were primarily in the innovation domain. Barriers included simulation sickness, headset discomfort, and limited immersive, interactive, and embodied experiences. Facilitators included technological advantages, highly immersive and interactive experiences, a safe training environment, individual user attributes, and structured orientation and support during training. VR training demonstrated benefits across multiple levels, from initial reactions and learning (knowledge, skills, and attitudes) to behavioral changes and systemic outcomes. This review highlights the current landscape of VR-based dementia care training. Future research should refine VR experiences and assess their impact on caregiver-resident interactions. Addressing barriers and leveraging facilitators can support the effective implementation of VR training to enhance care quality and resident well-being in long-term care settings.

